# Comment from the Editor on the Special Issue “Head and Neck Critical Illness: Basic and Clinical Research Implications”

**DOI:** 10.3390/jcm8111905

**Published:** 2019-11-07

**Authors:** Hiroyuki Tomita

**Affiliations:** Department of Tumor Pathology, Gifu University Graduate School of Medicine, 1-1 Yanagido, Gifu 501-1194, Japan; h_tomita@gifu-u.ac.jp

**Keywords:** head and neck cancer, NGS, squamous cell carcinoma

## Abstract

While oncogenic mutations of head and neck squamous cell carcinomas (HNSCC) in head and neck malignancies are uncommon, analysis using next-generation sequencing (NGS) technologies is growing. Further, single-cell analysis is being developed to overcome cancer cell heterogeneity and improve the poor survival of patients. However, it is important for researchers to know how to use this information to improve patients’ survival.

Among head and neck malignancies, head and neck squamous cell carcinomas (HNSCC) comprise a heterogeneous group of malignant neoplasms arising from the squamous cell epithelium of the upper aerodigestive tract. The sites of the HNSCC development include the oral cavity, nasopharynx, oropharynx, hypopharynx, and larynx. Squamous cell carcinomas arising from these sites account for the sixth most common malignancy worldwide [1]. The 5-year survival rates for HNSCCs have remained at approximately 50% for the past 40 years [2]. 

The understanding of the molecular and genetic alterations leading to oncogenesis in head and neck cancers, including HNSCC, has dramatically increased in the past decade. Initial steps taken to grasp the genetic pathogenesis of head and neck cancer paid attention to cytogenetic studies. The development of microarray technology has made it possible to classify HNSCCs into distinct types based on various gene expression patterns. Recently, next-generation sequencing (NGS) technologies have enabled many researchers to sequence a large number of cancers to identify novel gene abnormalities (i.e., mutations, translocations, and fusions). An underlying motivation for genomic profiling studies by these researchers was to gain a more radical understanding of the molecular alterations in head and neck cancer for the establishment of novel targeting therapeutics.

Conventional methods that have been used to study gene expression and chemotherapeutic responses in cancer molecular assays are performed on whole cell populations in tumor tissue. Therefore, the results from these methods average the differences between individual cells in tumor tissue [3]. This approach oversimplifies the complexity of the various genetic profiles existing in the tumor microenvironment (TME) and distorts results relating to the proportion and identity of cancer stem cells. 

On the contrary, single-cell genomic profiling by single-cell sequencing is performed independent of pooled samples or cell populations, permitting a higher fidelity representation of intra- and intertumoral cell heterogeneity in the TME [4,5]. Using single-cell sequencing means that each unique head and neck cancer cell type can be identified and elucidated. Further, a more profound discrimination of intra- and intertumoral differences is critical in developing novel therapeutic strategies targeted at increasing tumor-specific antigen responses [3]. 

Oncogenic mutations in HNSCC are uncommon. Targeting these alterations may require investigating comprehensive fatal approaches against cancer cells [5]. Moreover, oncogenic signaling pathways can be activated by various non-genetic mechanisms that are not detected by genomic efforts. It is necessary to challenge how we use the information obtained from NGS to develop and improve diagnostic and therapeutic modalities.
